# Selective Undercut of Undoped Optical Membranes for Spin-Active Color Centers in 4H- Silicon Carbide

**DOI:** 10.1021/acsnano.4c08702

**Published:** 2025-01-29

**Authors:** Jonathan Dietz, Amberly Xie, Aaron M. Day, Evelyn L. Hu

**Affiliations:** John A. Paulson School of Engineering and Applied Sciences, Harvard University, Cambridge, Massachusetts 02138, United States

**Keywords:** microfabrication, photoelectrochemistry, silicon carbide, color centers, thin films, electrochemical etching

## Abstract

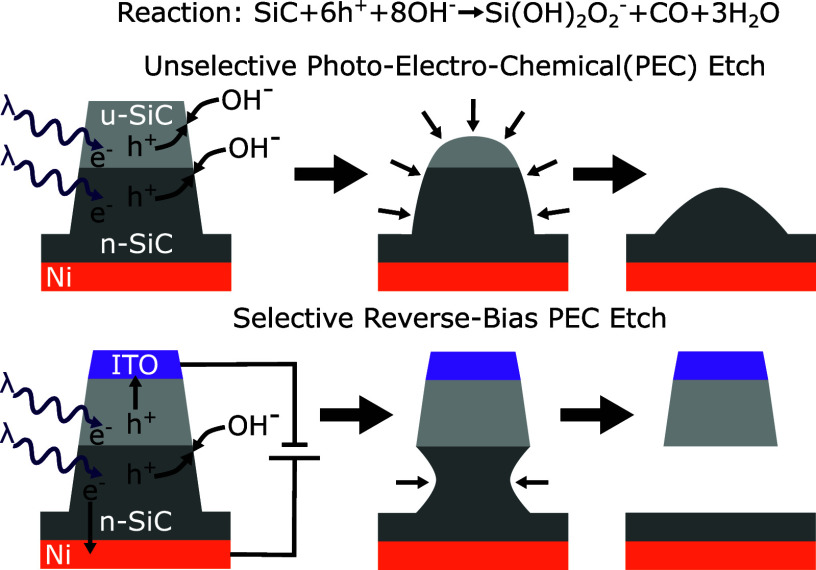

Silicon carbide (SiC) is a semiconductor used in quantum information processing, microelectromechanical systems, photonics, power electronics, and harsh environment sensors. However, its high-temperature stability, high breakdown voltage, wide bandgap, and high mechanical strength are accompanied by a chemical inertness, which makes complex micromachining difficult. Photoelectrochemical (PEC) etching is a simple, rapid means of wet processing SiC, including the use of dopant-selective etch stops that take advantage of the mature SiC homoepitaxy. However, dopant-selective PEC etching typically relies on highly doped material, which poses challenges for device applications such as quantum defects and photonics that benefit from low doping to produce robust emitter properties and high optical transparency. In this work, we develop a selective PEC process that relies not on high doping but on the electrical depletion of a fabricated diode structure, allowing the selective etching of an *n*-doped substrate wafer versus an undoped epitaxial (carrier density of 1(10)^14^ cm^–3^) device layer. We characterize the photoresponse and PEC behavior of the diode under bias and use those insights to suspend large (100 × 100 μm) undoped membranes of SiC. We further characterize the compatibility of membranes with quantum emitters, performing comparative spin spectroscopy between undoped and highly doped membrane structures, finding the use of undoped material improves ensemble spin lifetime by >5×. This work enables the fabrication of high-purity suspended thin films suitable for scalable photonics, mechanics, and quantum technologies in SiC.

## Introduction

A high-yield, systematic means of producing high-quality thin films is an outstanding challenge in realizing scalable quantum and classical technology. The availability of single crystal thin films with high purity and optical quality has led to large advances in miniaturizing typically bulk optical components with equivalent integrated photonic devices and the development of microelectromechanical circuits.^1−4^ One such approach to creating thin films in semiconductor materials with effective p and n-type doping is dopant-selective photoelectrochemical (PEC) or electrochemical etching of one doping type over the other in an epitaxial or implanted homojunction.^5^ Such etching strategies have been designed in silicon, gallium nitride, gallium arsenide, and silicon carbide homojunctions.^6−9^ However, these approaches rely on heavily doped device layers, as lowering the doping of the device layer reduces etching selectivity.^9^ A dopant-selective electrochemical etch that does not require high device layer doping is desirable in applications where high doping degrades performance or design flexibility, such as electronics, photonics, and mechanics or in material systems where effective doping is not available for either p-type or n-type, such as zinc oxide or diamond, respectively.^10−13^

This work presents such a process, with a particular application to Silicon Carbide (SiC), a wide band gap semiconductor widely studied for use in photonics, mechanics, and quantum information^14−17^ Consequently, SiC is such a material that could benefit from a PEC etching process that results in a low-doped thin film. For example, SiC hosts several solid-state quantum defect qubits, which show lifetime-limited optical line widths, long spin coherence, and high initialization fidelity, which could form the basis of quantum memory nodes in a quantum network.^18,19^ Dopant-selective PEC etching has successfully created suspended photonics in SiC with integrated defects, yet these devices and defects demonstrate degraded optical and spin performance, respectively, due to the required use of high doping concentration.^20−22^ This requirement is predicated on the ability to independently control band bending in each material doping type with respect to the chemical potential of decomposition in the etching electrolyte, which is more favorable in layers with large differences in doping.^23^

We present a modified PEC process that uses the bias of a simple transparent Shottky barrier diode to enable independent electrochemical control of each material layer. We show selective etching between an undoped layer of 4H–SiC and a highly doped substrate, demonstrating high etch rates (>4 μmh^–1^), low surface roughness (∼1 nm RMS), and large areas (>100 μm^2^) of undercut material. We further demonstrate the immediate device performance advantages of this process over a process that relies on high doping of the device layer alone, comparing the quantum spin performance of ensembles of silicon monovacancies in doped and undoped layers. When an undoped layer is used, lower strain, longer spin coherence, and lower inhomogeneous broadening are observed. The reliance of this technique on an externally fabricated diode device rather than solely on material properties indicates that this technique could be adapted to other material systems where junctions can be fabricated.

## Results/Discussion

Due to its highly chemically inert nature, suspended SiC photonic and mechanical devices are often created using ion-slicing, grind-and-polish, deep backside etching, or via dopant-selective PEC etching.^2,24−27^ These techniques have enabled fabrication of high-quality devices, but performance is typically limited by wafer nonuniformity, yield, excess strain, and charge noise. PEC etching usually relies on high doping contrast between epitaxial layers to selectively etch, for example, a highly n-doped substrate from a comparably p-doped epilayer, with the epilayer as the ultimate host of both photonic devices and quantum defects. However, free carrier absorption from highly doped material and charge noise creates optical propagation loss and limits qubit spin coherence.^22^ Previously fabricated devices demonstrated high-quality optical resonances, but suffered from high strain, roughened surfaces, and poor defect coherence.^22,28^

The PEC etching of single crystal SiC is well understood for both n and p doping types in alkaline solutions. In both cases, etching occurs through the elimination of silicon through the formation of a soluble silicate product and carbon monoxide via the charge exchange of holes with hydroxide ions in solution^29^
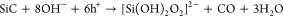
1

**Figure 1:**
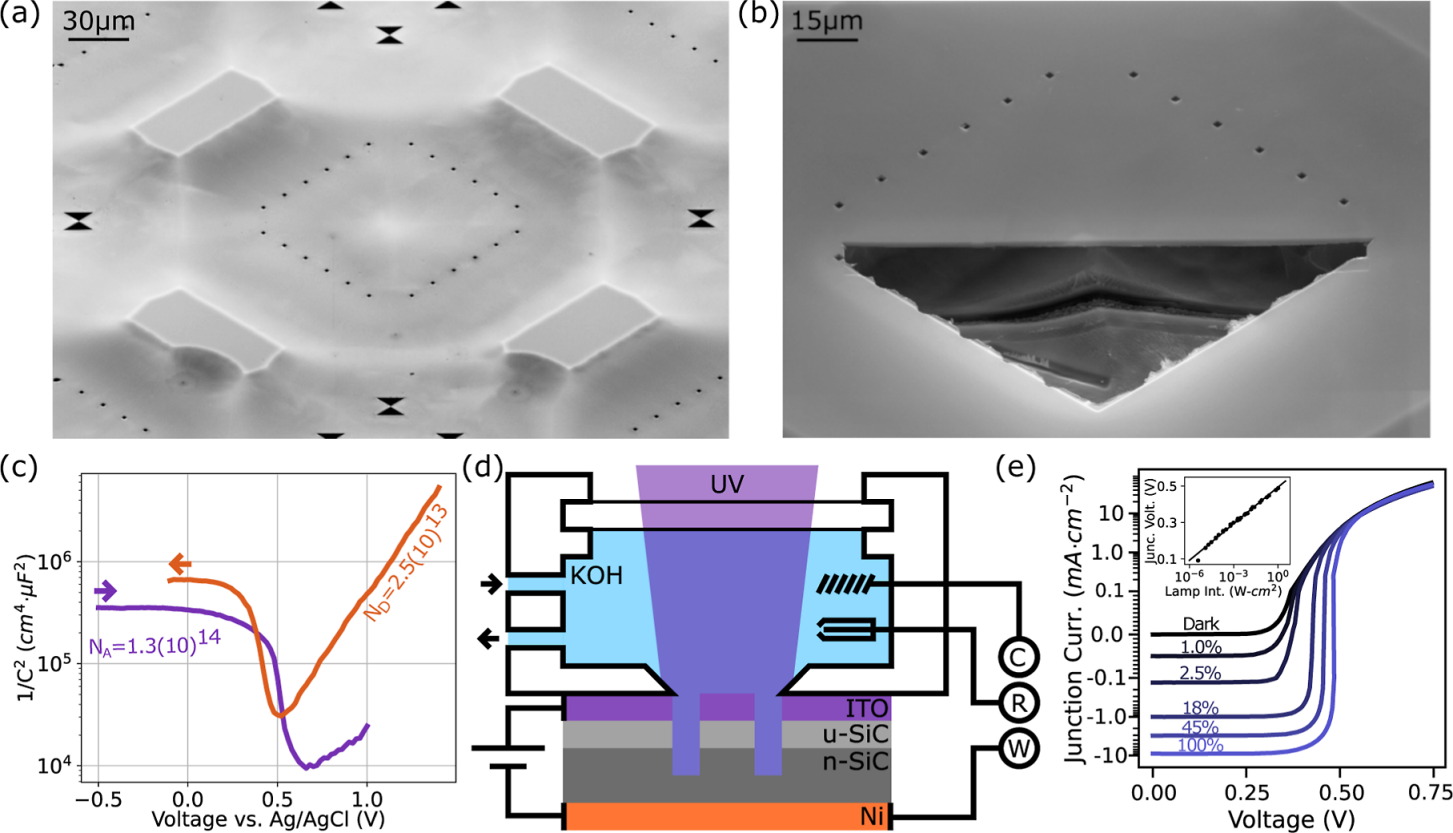
(a) Scanning electron microscopy image of a 100 × 100 μm membrane with support structures imaged at 30 keV to reveal the substrate structure. (b) Focused ion beam (FIB) cross section of membrane showing uniform membrane thickness. (c) Electrochemical impedance spectroscopy of an undoped, 500 nm thick epilayer of SiC showing low p-type conductivity, determined from linear fitting (1/*C*^2^) and measured using an Al contact. (d) Configuration of the PEC cell used to perform cyclic voltammetry (CV) and etching of 4H–SiC structures. *C*, *R*, and *W* are the Pt counter, Ag/AgCl reference, and SiC working electrodes, respectively. u-SiC denotes the unintentionally doped nature of the p^–^ layer. (e) *I*–*V* curves of the photodiode formed by the indium tin oxide (ITO) contacted undoped layer and Ni contacted substrate. Inset: illumination-dependent photobiasing of the diode.

In p-type material, electrochemical etching can occur without illumination because holes are a majority carrier in the material.^30^ Because the valence band in p-type material typically bends in energy downward toward the chemical potential of the electrolyte, the onset of etching occurs when the cell bias lowers the barrier holes experience in reaching this interface. By contrast, in n-type material, etching does not occur unless the SiC is illuminated to generate the requisite holes, and though the onset of etching can occur without cell bias, it is often beneficially controlled by the application of such a bias.^29^ Etching rate is limited by the rate of reaction at the semiconductor–electrolyte interface, controlled by the quantity and diffusion rate of ions in solution and holes in the semiconductor toward the interface. The diffusion rate of ions to the interface is controlled by mass transport in the bulk of the solution and the potential drop that ions experience at the Helmholtz double layer. In the semiconductor, the diffusion of holes to the interface is controlled by the built-in voltage of the semiconductor–electrolyte junction and the voltage difference between the semiconductor (working) electrode and the counter electrode. The number of holes is largely determined by the intensity of the illumination. PEC etching of silicon carbide is stable if anodic oxidation does not outpace the dissolution by charge exchange, in which case the etch is passivated by an insulating layer of SiO_2_.^29^ Additionally, the etching rate can be controlled by crystallography. In hexagonal polytypes of SiC, the carbon-face of the material is known to be significantly more reactive than the silicon-face of the material and consequently has a significantly faster PEC etch.^31^ p-type (n-type) material may be selectively etched against relatively n-type (p-type) material by appropriate selection of cell bias by setting a more negative (positive) cell bias which fixes the energy position of the valence band relative to the electrolyte‘s chemical potential, potentially yielding suspended structures if the substrate is targeted in the etch (Figure 1a–b). In a homojunction that combines both doping types, this relies on a high doping difference (e.g., *N*_*a*_ = 1(10)^18^ cm^–3^ vs *N*_*d*_ = 1(10)^18^ cm^–3^) and allows a 0.5 V potential window for selectively etching relatively thick layers of SiC.^23^

Unfortunately, in addition to the constraints on applications imposed by the high doping spread necessary for selective etching, doping selectivity in a homojunction depends on the ability to mutually bias both doping types in the cell. While this assumption is reasonable in thick layers or under low illumination conditions, it breaks down in epilayer thicknesses approaching useful thicknesses in photonic devices because of strong photobiasing of the homojunction. To assess this effect, we construct a junction from a thin, 500 nm undoped layer of 4H–SiC epitaxially deposited onto an n-type (3(10)^18^ cm^–3^ nitrogen doped) substrate wafer. The carrier concentration and conductivity type of the epilayer is determined using electrochemical impedance spectroscopy-based Mott–Schottky analysis^32^
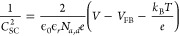
2where *C*_SC_ is the capacitance of the space charge region, ϵ_0_ and ϵ_*r*_ are the vacuum and relative (9.6 for 4H–SiC) permittivities, *N*_*a*,*d*_ is the acceptor or donor concentration, respectively. *e* is the electron charge, *k*_B_ is the Boltzmann constant and *T* is the temperature in degrees Kelvin. By linear fitting to the slope of the measured capacitance in cathodic and anodic sweeps, it is possible to determine the acceptor and donor concentrations, respectively. The net doping is found to be 1.15(10)^14^ cm^–3^, as shown in Figure 1c, hereafter referred to as the “p-type” layer.

For the PEC-enabled release of etched structures, ITO is adopted as an optically transparent contact to the p-type layer through which the underlying SiC can be illuminated while still providing uniform electrical connectivity across the cell. Before fabricating membrane devices, we first characterize the photoresponse of the diode structure, to determine the degree to which the epilayer is photobiased as the lamp intensity is varied. In the dark, the current–voltage (IV) curve shows a turn-on voltage of 0.5 V. Under increasing illumination, the diode shows a typical photodiode response, with a peak photobias of 490 mV measured at a lamp intensity of 1W·cm^–2^ (Figure 1e).

**Figure 2:**
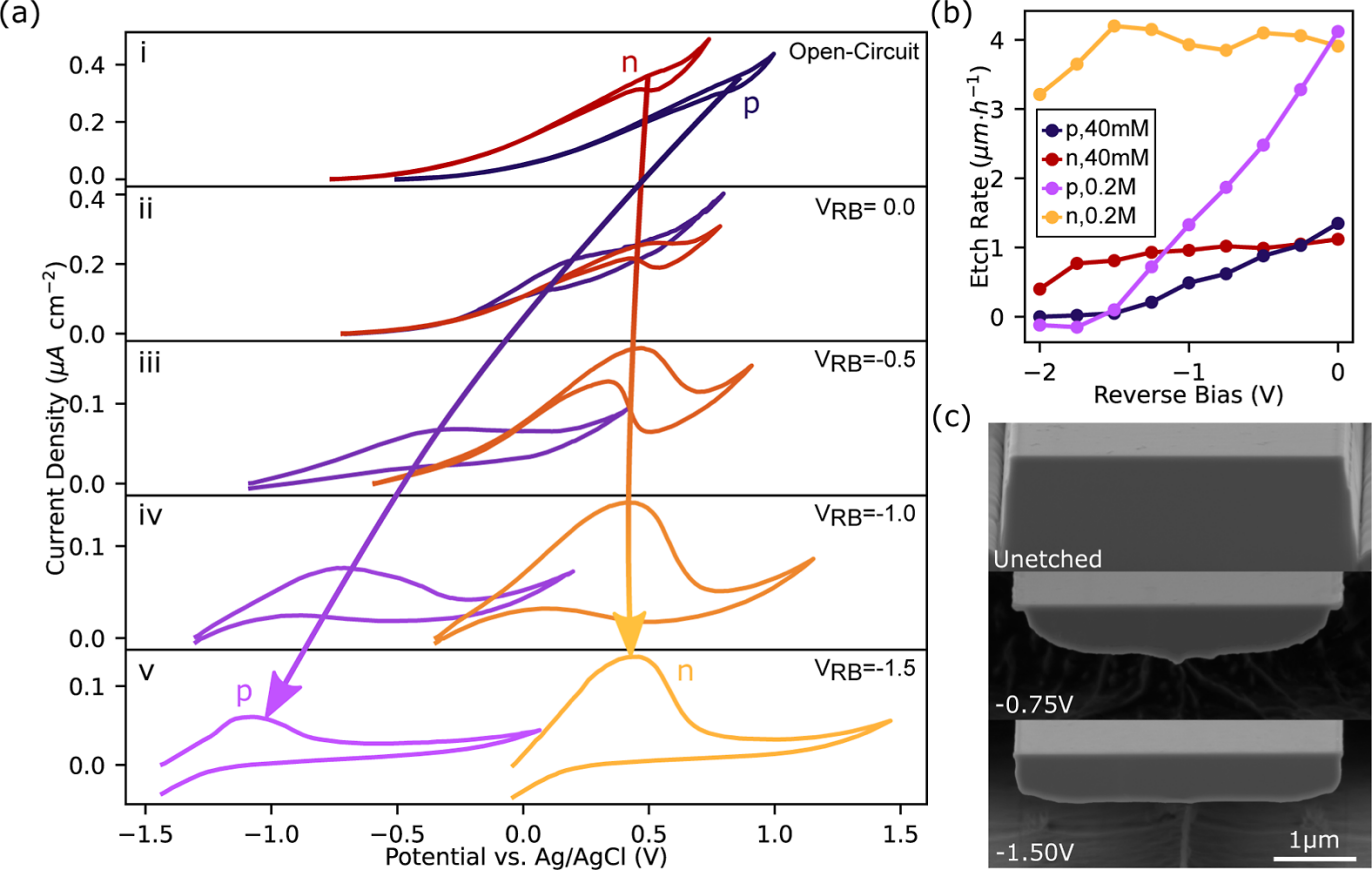
(a) Cyclic voltammograms (20 mV·s^–1^) of etched mesa structures under open circuit and various diode reverse biases, measured on the n-type contact (red, orange, and yellow) or p-type contact (indigo, purple). (b) Etch rates for different KOH concentrations as a function of diode reverse bias. (c) FIB cross section of mesa structures before PEC etching and after 2 h of PEC etching with a working electrode bias of 0.2 V and a partially depleted (−0.75 V) and fully depleted (−1.5 V) reverse bias.

To characterize the dopant-selective undercut process, several mesa type devices are fabricated using positive tone electron-beam lithography and plasma dry etching using ITO as a hard mask. ITO is found to be a highly selective hard mask, as oxide reduction is continually countered by plasma oxidation via the oxygen present in the SiC etching gas mixture, yielding a selectivity of 50:1 (see Methods). The membranes are undercut by using a PEC cell, with two separate electrical circuits present. The first is the potentiostat, used to bias either the n-type or p-type SiC as a working electrode against the Pt counter electrode. The second is the junction bias, controlled by attaching a voltage source to the p- and n-type contacts. To assess the impact of photobiasing on the etching kinetics of the junction, CV is performed under various junction bias conditions with a sweep rate of 20 mV·s^–1^ (Figure 2a). Under full-illumination (1W·cm^–2^) and no external bias applied to the junction, the p-type and n-type CV curves recorded are similar except that the p-type curve is shifted anodic relative to the n-type curve (Figure 2a.i). This is contrary to rigorous studies of thick layers of 4H–SiC, which found that n-type curves should be anodic relative to p-type curves.^23^ Furthermore, no distinct oxidation or reduction peaks are observed corresponding to p-type or n-type etching, confirming that the strong photobiasing of the cell has eliminated etch selectivity. However, by noting that the photobiasing occurs because of the presence of the junction formed by the epilayer and substrate, it can be concluded that the photobiasing can be completely suppressed by reverse biasing the junction to correct for the photogenerated voltage. By closing the junction circuit between the ITO and Ni junction contacts, the CV curves converge (Figure 2a.ii). While closing the junction circuit may restore selectivity in highly doped material, we must further increase the junction bias in our case of an undoped epilayer. The junction bias depletes the epilayer of carriers and creates selectivity between regions of different doping where it is not possible to achieve rapid selective etching without diode biasing. As greater reverse bias is progressively applied, the quasi-Fermi level in the valence band of the p-type layer rises relative to that in the n-type layer and the dissolution potential into the electrolyte, and the p-type CV curve shifts cathodically (Figure 2a.iii–v). At the same time, the characteristic oxidation and reduction peaks associated with controlled etching reappear now at cell biases significantly different enough to support selective etching.

To assess doping selectivity in realistic structures, a series of 4 μm wide mesas are undercut in 40 and 200 mM KOH at different junction reverse biases (Figure 2), with a cell bias of 0.2 V, selected to ensure that the n-type electrode is not passivated.^29^ Lateral etching rates and membrane morphology are determined using focused-ion-beam (FIB) cross sectioning. In both electrolyte concentrations, as the reverse bias increases, p-type etching is suppressed before becoming fully passivated, past a reverse bias of −1.3 V(Figure 2b). Cross-sectional images corroborate this finding, showing partial undercutting of the p-type layer at moderate reverse biases (−0.75 V), and full passivation at high reverse biases(−1.5 V) (Figure 2c). This result demonstrates that at sufficient reverse bias, diffusion of photogenerated carriers can be predominantly controlled not by differences in doping but by external biasing.

**Figure 3:**
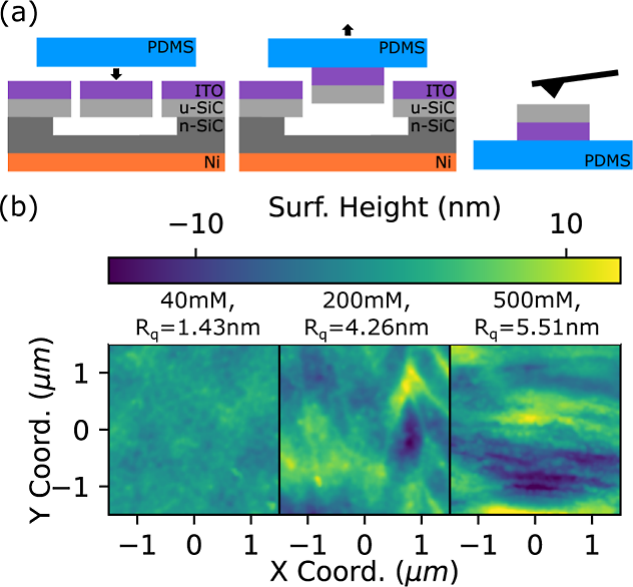
(a) PDMS membrane release process for AFM characterization. (b) AFM height maps for different electrolyte concentrations with corresponding root mean squared roughness values (*R*_q_) after a 2 h release process with 0.2 V cell bias and 1W·cm^–2^ illumination.

Smoothly etched surfaces are highly desirable for photonic and mechanical structures, as scattering loss and surface damping are therefore reduced. We investigate the impact that electrolyte concentration has on the surface roughness of the underside of our membranes. The membranes shown in Figure 2c are fully suspended and then lifted out using polydimethylsiloxane (PDMS) stamping (Figure 3a). The surface roughness of the membrane underside is then measured by tapping mode atomic force microscopy. We find that the concentration of electrolyte strongly controls the surface quality of the underside of the membranes, with lower concentration (40 mM KOH) yielding a RMS surface roughness value of 1.43 nm (Figure 3b–d), whereas higher concentrations of KOH yield rougher surfaces (5.51 nm for 500 mM KOH). Previous work suggests that roughness increases in a regime where oxide formation exceeds the rate of oxide removal, indicating that this balance may be more difficult to establish at high electrolyte concentrations.^23^ A similar work also showed that above 500 mM concentrations, only porous SiC is formed by PEC etching.^31^

To assess the potential of the material to host quantum spin registers, the reverse bias undercut process is paired with spin resonance and spin lifetime measurements. A suspended, 500 nm thick undoped membrane formed using the process described, etched in 40 mM KOH, is compared to another, 500 nm thick suspended membrane comprising p-i-p-n (150 nm p-type, 200 nm undoped, 150 nm p-type, n-type substrate) material used in previous work, which is undercut under identical conditions.^28^ Prior work found that slight shifts in the ZPL, optically detected magnetic resonance (ODMR), and spin coherence times in photoelectrochemical etched structures were, a result of high doping, porosity and strain induced by the PEC process.^22^ We select the silicon monovacancy for study as it can be flexibly introduced and has been shown to have high-quality optical and spin properties in nanostructures.^33,34^

**Figure 4:**
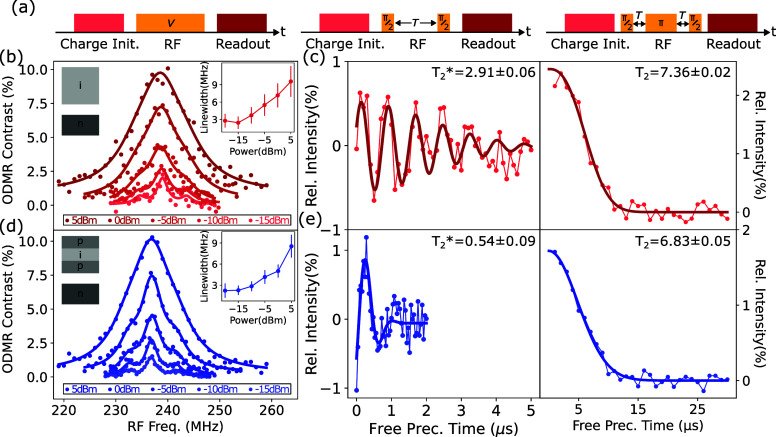
Spin spectroscopy of weak ensembles of *V*_Si_^–^ in (red) undoped epilayer and (blue) p-i-p doped structures. (a) Pulse sequences used for (i.) ODMR, (ii.) FID, and (iii.) spin dephasing measurements. (b-c) ODMR spectra with fitted triple Lorentzian spectrum based on natural ^29^Si concentration in the nearest neighboring site of the defects. Inset: Fitted power-dependent line-width of the central peak. (d–e) Free induction decay (*T*_2_*) fitted to a decaying sinusoid with an envelope of  measured via a Ramsey sequence 2 MHz off-resonance and spin dephasing time (*T*_2_) fitted to an exponential decay of  by Hahn echo sequence on-resonance. All measurements performed at 6K.

To compare the spin performance of emitters in both the heavily doped and undoped materials, power-dependent ODMR, free induction decay times (*T*_2_*), and spin dephasing times (*T*_2_) are measured for each ensemble (Figure 4a). The ground state (*S* = 3/2) spin levels, which are typically separated by a zero-field splitting of 70 MHz, are separated further by a moderate magnetic field of 60G to a final splitting of 240 MHz to reduce the effect of heteronuclear spin flip processes that limit the *T*_2_ and *T*_2_* times of ensemble of emitters. Assessing the  to  transition, ODMR studies in Figure 4b,c show similar performance, with emitters in both material types demonstrating narrow inhomogeneous broadening that saturate to a minimum at similar drive powers. The *T*_2_* time of 2.91 ± 0.06 μs measured in the undoped material (Figure 4d) is longer than that in the doped material (Figure 4e), approaching the theoretically predicted low field limit of (1.9 μs) for monovacancy spins in a naturally abundant ^29^Si, ^13^C nuclear bath.^35^ This demonstrates that the use of undoped material reduces the presence of influences that induce fast dephasing of the spins, which previously limited PEC-etched device performance.^22^ In contrast, the *T*_2_ time is similar between the two samples, showing that in both cases on longer time scales, the defects are limited by similar slow, dephasing processes. This implies that doping or material defects, and not material porosity or fabrication damage, primarily drive the degradation of *V*_Si_^–^ spin properties in PEC processed material.

## Conclusions

We have demonstrated a means of dopant-selective PEC etching that significantly relaxes constraints placed on doping density to achieve etch selectivity, thereby enabling the suspension of low-surface roughness, large-area membranes with an improved charge-noise environment to host quantum emitters. This approach reduces the epitaxial steps necessary for high purity, PEC-suspended photonics and mechanics in SiC to one step with the addition of a commonly used hard-mask (ITO) and a circuit to bias the junction formed between the epilayer and substrate. We investigate the photoelectrochemical attributes of SiC diodes under external biasing and show that smooth membranes of undoped silicon carbide can be suspended at rates exceeding 4 μm·h^–1^. This rate is likely limited by the rate of diffusion of ions from the electrolyte.^29,31^ We additionally note that the demonstrated 500 nm device layer is defined by epitaxial growth and can therefore be flexibly changed to suit application. This technique could potentially be used for the controllable transfer of SiC films to other substrates, for example, silicon oxide on silicon wafers, or used to precisely thin already bonded SiC material. Finally, we perform comparative spin spectroscopy on suspended doped and undoped materials, demonstrating that undoped material beneficially reduces the effect of fast spin noise on quantum defects and is compatible with PEC processing. The development of a means of reliably suspending large undoped membranes of SiC opens up new opportunities leveraging 4H–SiC’s high yield strength for nanomechanical oscillators and applications that integrate quantum emitters into electronic and photonic devices.^36,37^ Beyond SiC, this technique relaxes the material constraints for dopant selective etching, suggesting a means of selectively etching other inert materials where only a single doping type can be reliably created, such as diamond.

## Methods/Experimental

The material used in this study is purchased commercially from Pam-Xiamen Inc., specified as a 500 nm epilayer of undoped SiC on a 4° cut *c*-axis wafer. Contact is made to the n-type substrate by thermally evaporating 200 nm of nickel on the substrate backside followed by subsequent rapid thermal annealing for 5 min at 950 °C in an argon atmosphere.

For determining the carrier concentration of the epilayer, aluminum is thermally evaporated onto the epilayer, with a circular 2 mm aperture in the center of the sample left masked. The mask is then stripped to expose the underlying undoped SiC epilayer, and contact is made by rapid thermal annealing for 5 min at 950 °C in an argon atmosphere. The sample is then immersed in 2 M KCl solution with the Al contact covered by a silicone O-ring.

For PEC etching and CV, a transparent top electrode of indium–tin oxide (ITO) was sputter deposited at a rate of 3 nm·min^–1^ to form a 100 nm layer, ramping the substrate to 600 °C over the course of deposition. Substrate heating was found to be necessary to form a suitably conductive contact with the p-type epi-layer. Illumination for all measurements and etching is provided by a 500 W Xe–Hg Arc lamp with its visible spectrum filtered by a 400 nm short-pass filter to reduce excess heating of the sample, producing a peak intensity of 1W·cm^–2^. For membrane fabrication, mesas are first defined by patterning the ITO layer using positive tone (PMMA495 C6) electron-beam lithography (Elionix F125 operated at 150 keV, 2 nA). The pattern is transferred to the ITO by inductively coupled plasma reactive ion etching (ICP-RIE) in 3 parts H_2_:1 part CF_4_ for 3 min, and then subsequently transferred into the SiC to a depth of 2 μm using ICP-RIE in 4 parts SF_6_:1 part O_2_ for 10 min.

PEC etching is performed in 40–500 mM potassium hydroxide (KOH) electrolyte, which is continuously circulated from a 10 °C reservoir to ensure a constant electrolyte concentration and to extract waste heat, with no supporting electrolyte added. The counter electrode is a wound Pt wire, and the reference electrode is an Ag/AgCl electrode in 3 M KCl.

For spin measurements, each sample is identically implanted with He ions of energy 37 keV at a dose of 10^11^ions·cm^–2^ followed by quenching from 650 °C after annealing in air for 30 min, to form a weak ensemble of silicon monovacancies (*V*_Si_^–^). The samples are then loaded into a 4K flow cryostat and fitted with a microwave wire antenna to excite ground state spin resonances. A ring magnet is placed aligned with the barrel of the microscope objective in order to align the axis of the magnetic field with the *c*-axis of the crystal, before being measured by a gaussmeter as 60 G. The charge state of the defects is initialized by an off resonant helium neon laser, and photoluminescence is measured on the phonon sideband (>925 nm) of the defects using near resonant excitation with a 916 nm external cavity diode laser with a power of 100 mW, switched by acousto-optic-modulators (AOM) coupled to optical fibers to improve switching extinction. Ensembles are initialized with a power of 3 mW for 10 μs followed by a 1 μs wait time to allow the AOM to switch off.

For ODMR, The defects are then excited by microwaves pulses that are 50 μs long followed by a 5 μs resonant read-out laser pulse (signal *S*). An equal length sequence is then completed without microwave driving, followed by another read-out pulse (background *B*). For *T*_2_* and *T*_2_ measurement, the same initialization is performed followed by short microwave pulses of length τ_π_ = 250 ns (Figure 4a). In this case, the defects are read-out by a 1 μs resonant laser pulse (signal *S*). An equal length sequence is then completed with a single 180° shifted τ_π/2_ pulse, followed immediately by a read-out pulse (background *B*). The lifetime is determined by the duration of time between pulses in the signal shot, that is, the falling edge of each microwave pulse and the rising edge of the subsequent pulse. For all measurements, ODMR contrast or relative intensity is then determined by dividing background from signal: .
